# Immunohistochemical changes in rat retinas at various time periods of elevated intraocular pressure

**Published:** 2009-12-10

**Authors:** María Hernandez, F. David Rodriguez, S.C. Sharma, Elena Vecino

**Affiliations:** 1 Department of Cell Biology and Histology, University of the Basque Country, Leioa, Vizcaya, Spain; 2 Department of Biochemistry and Molecular Biology, University of Salamanca, Salamanca, Spain; 3 Department of Ophthalmology and Cell Biology and Anatomy, New York Medical College, Valhalla, New York, NY

## Abstract

**Purpose:**

To study alterations in different retinal cell types associated with retinal ganglion cell (RGC) death after elevation of intraocular pressure (IOP) in rats.

**Methods:**

IOP was elevated by episcleral vein cauterization of the rat left eye. The right unoperated eye was kept as the control. IOP was measured when rats were awake. The animals were euthanized after one week (n=4) and five weeks (n=4). Their eyes were enucleated, postfixed, cryoprotected, and embedded in optimal cutting temperature (OCT) medium. Cryosections of the retina were cut at 14 µm thickness and processed for immunocytochemistry with 15 antibodies that specifically stain different retinal cell types. The distribution and intensity of the label was analyzed by comparing sections of control and glaucomatous retinas obtained from identical locations.

**Results:**

The amount of amacrine cells identified by calcium binding proteins and choline acetyltransferase antibodies decreased after five weeks of elevated IOP. By using the anti-protein kinase C-α antibody, we were able to label a subpopulation of rod bipolar cells in control retinas but not in retinas that had elevated IOP. No changes were found in RGCs labeled with brain derived neurotrophic factor when comparing control and glaucomatous retinas. Glial fibrillary acidic protein and vimentin expression in glial cells increased after one week of elevated IOP.

**Conclusions:**

After one week of elevated IOP and before the onset of RGC death, it was evident that inner retinal cells showed remarkable changes in their molecular expression.

## Introduction

Glaucoma is an optic neuropathy characterized by the elevation of intraocular pressure (IOP) that leads to degeneration of the axons and somas of the retinal ganglion cells (RGCs). Clinical studies have demonstrated that a decrease in IOP is associated with the attenuation of retinal damage. However, after successful treatment that lowers IOP there is a continuation of visual field loss in some individuals [1–3]. Studies performed in rats have shown that neuroprotection of the retina is feasible with a small reduction of IOP [4–6].

Experimental glaucoma studies have mainly focused on RGC damage [7–10]. However, the cells that directly (amacrine and bipolar cells) or indirectly (photoreceptor and horizontal cells) come in contact with RGCs may also be damaged. Hence, cells in the inner retina may be affected in glaucoma as well as following ischemic damage.

Functional electroretinographic (ERG) studies have provided clear evidence of retinal damage. In glaucomatous retinas, ERG changes involve both the a-waves and b-waves [11,12]. Previous studies performed in rats with episcleral vein cauterization showed that an increase in oscillatory potentials (OPs) appeared before any morphological changes were seen in RGCs. OPs are generated by bipolar and amacrine cells localized in the inner nuclear layer (INL) [12]. Changes in a-waves and b-waves in glaucomatous retinas returned to control conditions when the IOP was lowered to basal values [13,14]. Also, an increase in the amplitude of the OPs was present in glaucomatous animals [15].

Several electrophysiological studies support the notion that the b-wave component of the ERG is generated by the interaction between the photoreceptors and the “on” bipolar cells. Depolarization of bipolar cells is the primary event in the generation of the ERG b-wave [16]. A decrease in the amplitude of the b-wave in the ischemic retina has been reported. Also, a disturbance of the retinal calcium homeostasis induced by high levels of excitatory amino acids was seen [17–19]. The observed changes may be a consequence of the perturbation of the retinal connections that we try to study at the morphological level.

The functional alterations observed in glaucoma as well as in ischemic conditions may reflect biochemical and immunohistochemical changes. Recent studies have shown that bipolar to amacrine cell signaling was altered in retinal ischemia and reperfusion experiments. However, immunohistochemical labeling of the neurons did not correspond to the functional deficits seen [20,21].

Following ischemia-reperfusion and optic nerve injury, immunocytochemical altered patterns in amacrine cells were reported in rabbit [18] and rat retina [22,23]. Similar changes were seen in animals with elevated IOP [24]. In glaucomatous animals, the reduction in amacrine cell number appears to be attributable to the loss of GABAergic, cholinergic as well as nitric oxide synthase (NOS) subpopulations. In another study, no significant changes were detected in the number of amacrine cells following elevation of IOP, but a loss of GABA and glycine labeling after optic nerve transection was reported [25]. The discrepancy observed in the results relating the extent of damage of amacrine cells after elevation of IOP may be due to the different methodologies used to increase IOP and to evaluate the changes, as well as the time periods studied. The main purpose of this study was to establish, by using a variety of cell-specific markers, whether neuronal degeneration or changes in the inner retina occurred in a rat model of experimental glaucoma.

## Methods

### Animals and tissue fixation

Eight adult female Sprague-Dawley rats bred in our university animal house (University of the Basque Country (UPV/EHU). Rats weighing 250 g were used throughout the study. Animals were housed in a room with a 12 h:12 h light-dark cycle, constant temperature (21 °C), and food and water ad libitum. At the indicated times (1 week and 5 weeks of elevated IOP), animals were anesthesized and perfused with 4% paraformaldehyde in 0.1 M phosphate buffer (PB, pH 7.4). To achieve the correct orientation of the eye during sectioning, before eye enucleation, we labeled the dorsal part of the eye with a permanent marker. We made an incision in the cornea to allow the fixative, sucrose, and optimal cutting temperature (OCT) medium to penetrate into the anterior chamber. Afterwards, the whole eyes were embedded in OCT compound in liquid nitrogen.

The eyecups were post fixed in the same fixative for 2 h at room temperature, cryoprotected for 24 h in 30% sucrose in 0.1 M PB at 4 °C, and embedded in OCT medium. Next, 14 µm thick cryosections were cut and stored at −20 °C until used. All animal experiments adhered to the ARVO Statement for the use of Animals in Ophthalmic and Vision Research.

### Elevation of IOP (episcleral vein cauterization)

A rise in IOP was induced by cauterizing three episcleral veins in the left eye, according to the method previously described [26,27]. Briefly, two dorsal episcleral veins, located near the superior rectus muscle, and one temporal episcleral vein, situated close to the lateral rectus muscle were isolated from the adjacent tissues. Cauterization was performed with a low-temperature cautery (500391, World Precision Instrument, Inc., Sarasota, FL) precisely applied to the selected vein. Special care was taken so that thermal damage to the neighboring tissues was avoided. The unoperated right eyes were used as control eyes throughout this study. Animals were divided in two groups: group 1 contained animals whose retinas were fixed after one week of elevated IOP, and group 2 included animals who were euthanized after five weeks of elevated IOP. Euthanasia was performed by an intraperitoneal injection of xylazine (Rompun; Bayer, SA, Barcelona, Spain) and ketamine hydrochloride (Ketolar; Parke-Davis, SL, Barcelona, Spain; 7.4 mg/ml and 31.5 mg/ml, respectively). Afterwards, the animals were perfused with 4% paraformaldehyde in 0.1 M phosphate buffer saline (PBS, pH 7.4). Using an applanation tonometer (TonoPen XL; Mentor, Norwell, MA), we measured IOP daily in the first group and weekly in the second group. This was done at the same time (3 PM) to avoid circadian IOP changes [27]. The results of the IOP reading were accepted if the confidence interval was greater than or equal to 95%. The mean values of the IOP measurements were eventually averaged, and results were expressed as mean±SEM. Statistical significance was established by Student *t*-test.

### Immunohistochemistry

Fifteen antibodies were used to identify and define different cell types or layers of the retina (Table 1). Retinal sections used for specific antibody labeling were collected from the central retina, both dorsal and ventral to the optic nerve. For the sake of consistency, photographs were always taken (on both sides of the optic nerve head) at half the distance between the center of the head of the optic nerve and the periphery of the retina. In this manner, we have analyzed antibody reactivity patterns at an identical location of the retina in all samples. The immunocytochemical reaction was performed simultaneously for control, one week, and five weeks glaucomatous retinal sections for each antibody. Two slides, containing sections from an identical localization, were processed for each experimental group. Negative control of the immunohistochemistry included incubation with nonimmune serum.

**Table 1:** List of primary antibodies used in this study

**Antigen**	**Host**	**Source, catalog number**	**Working concentration**	**Localization**
M/L cone opsin	Rabbit polyclonal	Chemicon, AB5405	1:10,000	OS of M/L cones
S cone opsin	Rabbit polyclonal	Chemicon, AB5407	1:5,000	OS of S cones
Bassoom	Mouse monoclonal	Abcam, 13249	1:200	Neuron synapses, OPL and IPL
Calbindin	Rabbit polyclonal	Swant, CB-38a	1:1,000	Horizontal and amacrine cells
Parvalbumin (PV)	Rabbit polyclonal	Swant, PV-28	1:2,000	Amacrine cells
Calretinin	Rabbit polyclonal	Sigma, C7479	1:1,000	Amacrine cells and subpopulations of RGCs
Calretinin	Mouse monoclonal	Swant, CR-39	1:1,000	Amacrine cells and subpopulations of RGCs
Choline acetyltransferase (ChAT)	Goat polyclonal	Chemicon AB144P	1:500	Cholinergic amacrine cells and IPL
Tyrosine hydroxylase (TH)	Rabbit polyclonal	Chemicon, AB152	1:500	Dopaminergic amacrine cells
Goα	Mouse monoclonal	Chemicon, MAB3073	1:50	“on” bipolar cells
Protein Kinase C-α (PKC- α)	Mouse monoclonal	Santa Cruz, SC-208	1:2,000	Rod bipolar cells
Protein Kinase C-α (PKC- α)	Rabbit monoclonal	Santa Cruz, SC-8393	1:100	Rod bipolar cells
Brain Derived Neurotrophic Factor (BDNF)	Rabbit polyclonal	Santa Cruz, SC-546	1:500	Amacrine cells and subpopulations of RGCs
Vimentin	Mouse monoclonal	Dako, M0725	1:2,000	Müller cells and astrocytes
Glial Fibrillary Acidic Protein(GFAP)	Rabbit polyclonal	Dako, Z0334	1:1,000	Astrocytes

Detailed information is given about the antibodies used, the host, the commercial source and catalog number, as well as the working concentration and its localization in rat retina. Abbreviations: BDNF represents Brain Derived Neurothrophic factor, CB represents calbindin, ChAT represents Choline acetyltransferase, CR represents Calretinin, GFAP represents glial fibrillary acidic protein, PKC represents protein Kinase C, TH represents tyrosine hydroxilase, IPL represents inner plexiform layer, OPL represents outer plexiform layer, OS represents outer segment, RGCs represents retinal ganglion cells.

Sections were incubated in a solution that contained 0.1 M Phosphate Buffer Saline (PBS; pH 7.4) and 0.25% Triton-X 100 (Sigma-Aldrich, St. Louis, MO) two times for 10 min at room temperature to improve tissue permeability. The retinas were incubated with primary antibody for 24 h at 4 °C. The primary antibody was diluted in 1% BSA in PBS solution. After incubation, sections were washed twice in PBS for 10 min each time. Then, retinas were incubated for 1 h with 1:200 goat anti-mouse IgG/Texas Red (monoclonal antibody; Molecular Probes, Portland, OR) or 1:200 goat anti-rabbit IgG/Bodipy FL (polyclonal antibody; Molecular Probes). Cell nuclei were stained with 1 μg/ml 4,6-diaminodiphenyl-2-phenylindole (DAPI; Sigma) in PBS and incubated together with a fluorescent secondary antibody. Finally, the slides were washed three times with PBS and mounted in 1:1 PBS and glycerol (by volume). All preparations were observed under an Axioskop 2 epifluorescent microscope (Zeiss, Jena, Germany) and photographed using a Coolsnap digital camera (RS Photometrics, Tucson, AZ). Excitation/emission wavelengths were 515/513 nm (Bodipy FL, Eugene, OR) and 595–615 nm (Texas Red, Eugene, OR).

Confocal microscopy (Olympus FV 500) was performed to verify the presence of double-fluorescent label in bipolar and amacrine cells. Confocal pictures obtained as stacks of images (1 μm thickness) were analyzed with the confocal software (Olympus, Tokyo, Japan). Image processing was done electronically (Photoshop; Adobe Systems, Mountain View, CA).

### Quantification of labeled cells

We have quantified the labeled cells for the following markers: opsins (M, L, and S), which labels the outer segment (OS) of the cone photoreceptors, parvalbumin (PV), which labels AII amacrine cells, choline acetyltransferase (ChAT), which labels cholinergic amacrine and ganglion cells, Protein Kinase-C-α (PKC-α) which labels rod bipolar cells and Goα which labels “on” bipolar cells. For each experimental group, at least ten images at 40× were used to count the immunolabelled cells. An unoperated contralateral retina from the same animal was used as control. The pictures were obtained always from the central retina, dorsal and ventral to the optic nerve head. Immunolabeled cells were counted by following the method used by Kielczewski et al. [25]. Briefly, three cryosections from each control and glaucomatous rat eye, were immunostained for the different antibodies. Ten images from each section were taken under 40× magnification. The linear distance of each picture was measured (Axiovision 4.7; Zeiss) and thus the number of positive cells was expressed as labeled neurons per linear millimeters of retina. Thus, the number of positive cells was expressed as a mean of labeled cells per mm. For statistical analysis, the Student *t*-test was applied.

## Results

### Intraocular pressure

**Figure 1:**
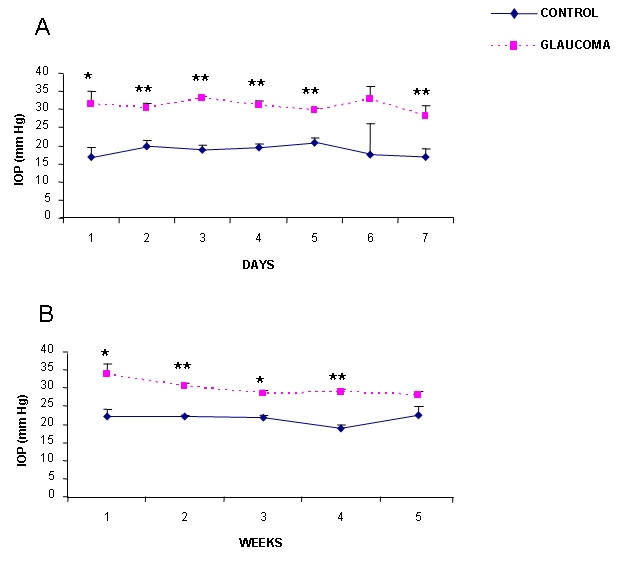
Time-course changes in intraocular pressure (IOP) after experimental glaucoma. Panel **A** shows the changes in IOP (mmHg) in group 1 (1 week of elevated IOP). Panel **B** shows the changes in IOP (mmHg) in group 2 (5 weeks of elevated IOP). Blue dots represent control values measured in the right eye, and purple dots represent experimental values measured in the left operated eye. The results of the IOP reading were accepted if the confidence interval was greater than or equal to 95%. The mean values of the IOP measurements were eventually averaged, and results were expressed as mean IOP±SEM. Five such measurements were made. Statistically significant differences were assessed according to Student’s *t*-test. Asterisks denote the level of significance as follows: *p≤0.05 and **p≤0.01.

In group 1 (one week of elevated IOP) mean control basal IOP during the first week of this study was 18.6±0.6 mmHg (mean±SEM). IOP fluctuated between 16.8 and 20.8 mmHg. In the glaucomatous left eye a significant increase in IOP was measured leading to a mean IOP of 30.9±0.6 mmHg. It oscillated between 29.6 and 33.0 mmHg (statistical significance p<0.01 when compared with control values; Figure 1A). In group 2 (five weeks of elevated IOP), the mean basal IOP of the control right eye was 21.5±0.6 mmHg (values between 18.9 and 22.5 mmHg) and that of glaucomatous left eye was 29.8±1.0 mm (values ranged between 28 and 33.6 mmHg (Statistical significance, p<0.01; Figure 1B).

### Immunohistochemistry

Specific antibodies that labeled identifiable retinal cells were used throughout. For details, see Table 1.

### Photoreceptor layer

**Figure 2:**
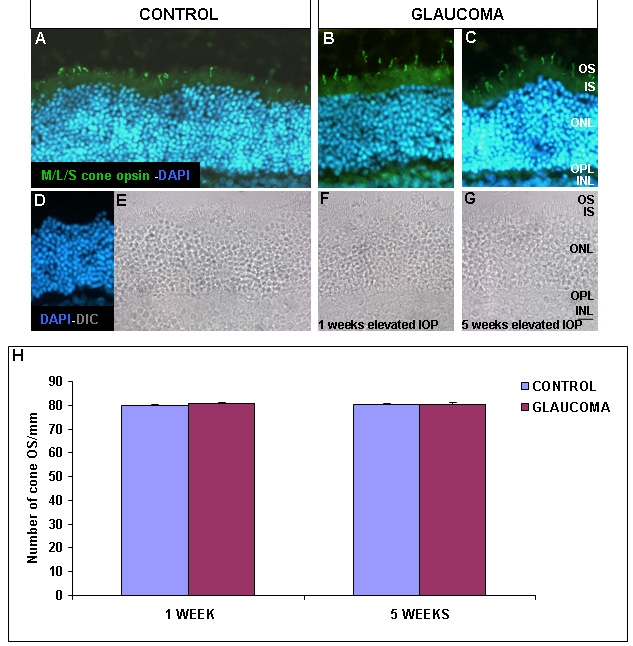
M/L and S cone opsin immunohistochemical localization. Control (**A**, **D**, and **E**) and glaucomatous retinas (**B**, **C**, **F**, and **G**) were labeled with M/L and S cone opsin. Photographs **B** and **F** represent retinas after 1 week of elevated IOP and photographs **C** and **G** show retinas after 5 weeks of elevated IOP. Panel **H** represents the number of cone OS/mm in control and experimental groups as indicated (n=40 in all experimental groups). Abbreviations: inner nuclear layer (INL), intraocular pressure (IOP), inner segment (IS), outer nuclear layer (ONL), outer plexiform layer (OPL), outer segment (OS), Differential Interference Contrast (DIC), 4,6-diaminodiphenyl-2-phenylindole (DAPI). Scale bar equals 20 μm.

Cellular markers were used to tag subtypes of cells in the OS of the outer nuclear layer. The cone outer segment was identified by using Middle and Long wavelength cones (M/L) opsin antibody that labels middle and long wavelength cones. The OS of the short wavelength cones was labeled with Short wavelength cones (S opsin) antibody. These antibodies show the same labeling patterns in control (Figure 2A) as in the glaucomatous retinas (Figure 2B,C**).** Moreover, cone outer segments were localized with Differential Interference Contrast (DIC) image at a similar location in the control (Figure 2E) as well as in the glaucomatous retinas (Figure 2F-G). There are no differences in the number of the M/L and S cone OS per mm when comparing control and both experimental groups (Figure 2H).

### Horizontal and amacrine cells

**Figure 3:**
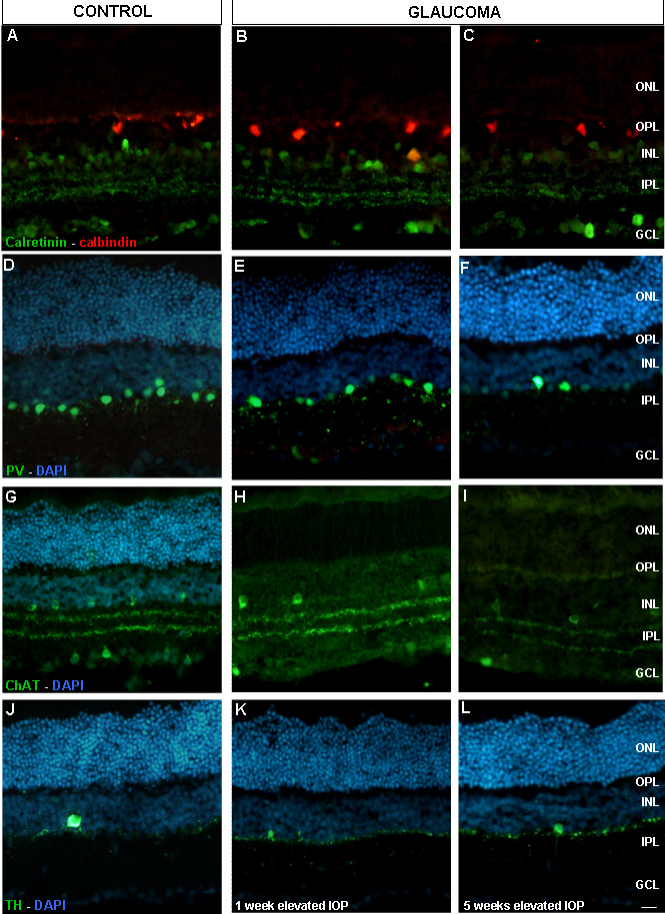
Calretinin, calbindin, parvalbumin, Chat, and TH inmunolocalization. Panels **A**-**C** show caretinin (green) and calbindin (red). Panels **D**-**F** show parvalbumin (green) and DAPI (blue). Panels **G**-**I** show ChAT (green) and DAPI (blue). Panels **J**-**L** show TH (green) and DAPI (blue). Pictures **A-J** represent control retinas, pictures **B**, **E**, **H**, and **K** represent glaucomatous retinas from experimental group 1 and pictures **C**, **F**, **I**, and **L** show glaucomatous retinas from experimental group 2. Abbreviations: choline acetyltransferase (ChAT), ganglion cell layer (GCL), inner nuclear layer (INL), intraocular pressure (IOP), inner plexiform layer (IPL), outer nuclear layer (ONL), outer plexiform layer (OPL), parvalbumin (PV), tyrosine hydroxylase (TH). Scale bar equals 20 μm.

No changes were found in horizontal cells labeled with calbindin D-28 kDa antibody either in the control or glaucomatous retina (Figure 3A-C). Many populations of amacrine cells were labeled with calretinin antibody (Figure 3A-C) while PV antibody labeled A-II amacrine cells (Figure 3D-F). Calretinin positive cells were localized in the outer and inner portion of the INL, as well as in the GCL. However, PV-positive cells were localized in the innermost portion of the INL.

One week after IOP elevation, calretinin positive amacrine cells showed a similar pattern compared to the control retinas. However, after five weeks of elevated IOP an appreciable decrease in the labeling, both in calretinin (Figure 3C) and PV-positive cells (Figure 3F) were observed.

**Figure 4:**
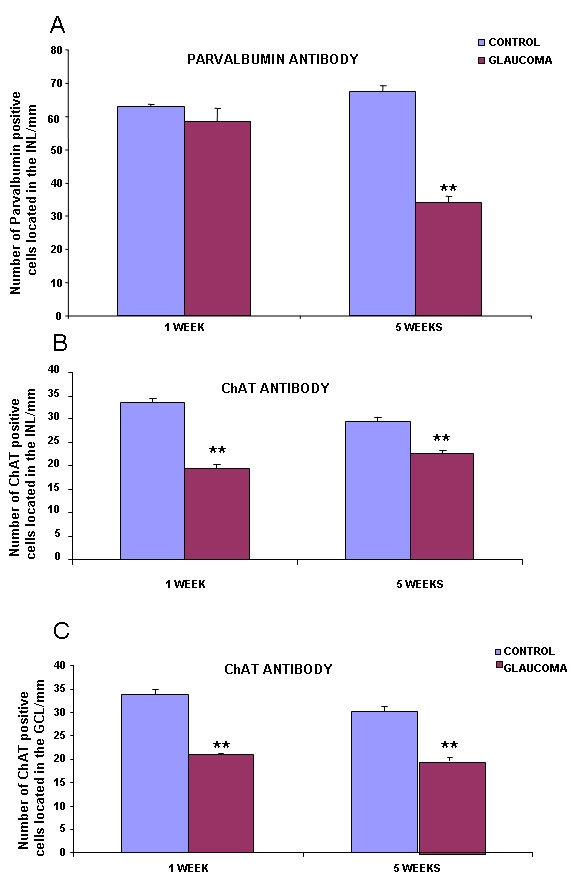
Number of cells/mm labeled with parvalbumin and ChAT antibodies. Panel **A** depicts the number of amacrine cells, in the inner nuclear layer, labeled with parvalbumin. Panel **B** shows the number of amacrine cells, in the inner nuclear layer, labeled with ChAT. Panel **C** shows the number of ChAT positive cells localized in the ganglion cell layer (n=40 in all experimental groups). Statistically significant differences were assessed according to Student’s *t*-test. Asterisks denote the level of significance as follows: *p≤0.05 and **p≤0.01. Abbreviations: choline acetyltransferase (ChAT), ganglion cell layer (GCL), inner nuclear layer (INL).

Positive PV amacrine cells were counted as described before. No statistical differences between control and glaucomatous retinas, after one week of elevated IOP, were seen. However, after five weeks of elevated IOP, we observed a significant decrease in amacrine cells labeled with PV (Figure 4A).

Control amacrine cells labeled with ChAT antibody were localized in the IPL and GCL. After one and five weeks of elevated IOP, there was a reduction in the number of marked cholinergic amacrine and ganglion cells (Figure 3G-I and Figure 4B,C**)**.

Amacrine cells labeled with tyrosine hydroxylase (TH) antibodies were scarce. No differences were found in the number of cells labeled after one and five weeks of elevated IOP (Figure 3J-L**).**

### Bipolar cells

**Figure 5:**
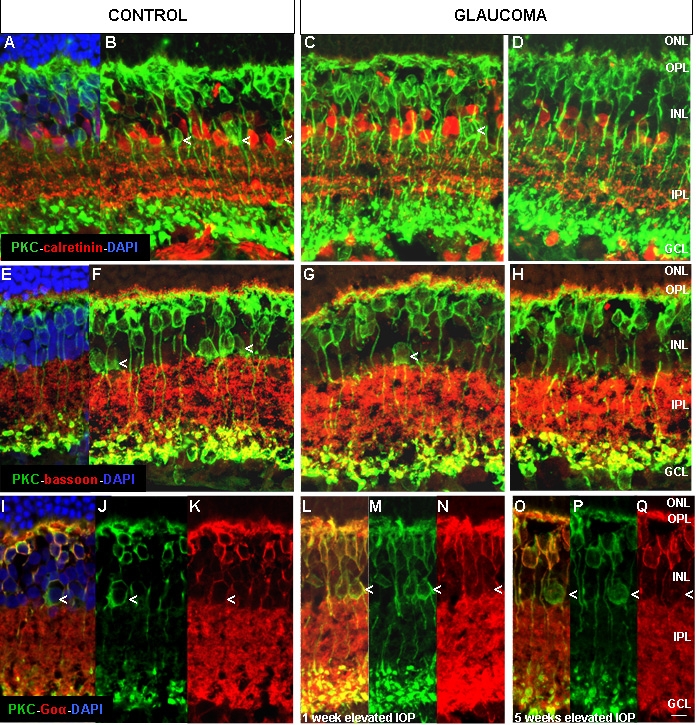
Confocal images of PKC-α, calretinin, bassoon, and Goα immunolocalization. Panels **A**-**D** show PKC-α (green), calretinin (red) and DAPI (blue). Panels **E**-**H** show PKC-α (green), bassoon (red), and DAPI (blue). Panels **I**-**Q** show PKC-α (green), Goα (red), and DAPI (blue). Arrows in **B**, **C**, **F**, **G**, and **I**-**Q** indicate a subpopulation of PKC-α-positive cells, located in the innermost INL, that may be affected by glaucoma. Abbreviations: ganglion cell layer (GCL), inner nuclear layer (INL), intraocular pressure (IOP), inner plexiform layer (IPL), outer nuclear layer (ONL), outer plexiform layer (OPL), protein kinase C-α (PKC-α). Scale bar equals 20 μm.

Bipolar cells were tagged with PKC-α and Goα antibodies. PKC-α in the control retina was present in the outer and inner parts of the INL (Figure 5). To identify if the cells located in the inner part of the INL were PKC-α-positive amacrine or bipolar cells, we double labeled with PKC-α and calretinin antibodies. Calretinin was present in amacrine cells but did not colocalize with PKC-α bipolar cells (Figure 5A-D). After one and five weeks of elevated IOP, there was a reduction in the number of PKC-α immunoreactive cells in the innermost portion of the INL (Figure 5C-D).

Bassoon antibody marks synaptic ribbons in OPL and conventional synapses in the IPL. Therefore, it has been used in the present study to define the limits between the IPL and INL, where PKC-affected cells by glaucoma were detected. No evident synaptic changes were found between the control and glaucomatous retinas by using bassoon (Figure 5E-H).

PKC-α is present in rod bipolar and a subpopulation of amacrine cells, while Goα is present in both rod bipolar and “on” cone bipolar cells. Since we have found a subpopulation of PKC-α immunoreactive cells in the innermost portion of the INL that were affected by the increase of IOP, we double labeled with both PKC-α and Goα antibodies to identify this subpopulation (Figure 5I-Q).

**Figure 6:**
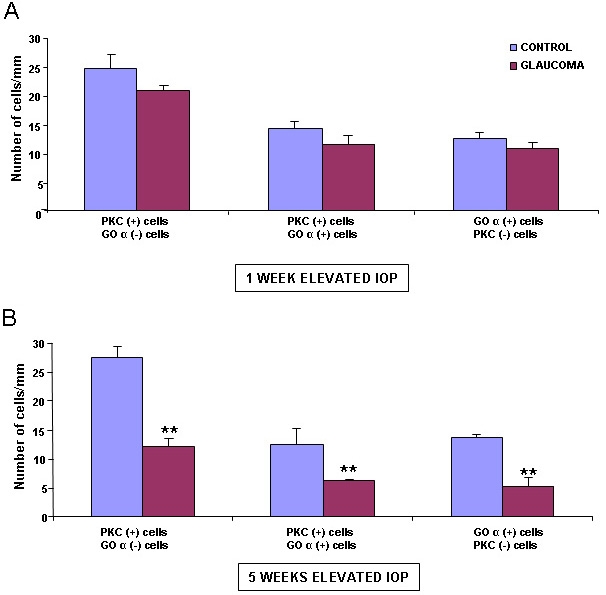
Number of cells per mm located in the INL labeled with PKC-α and Goα antibodies. (**A**) corresponds to one week of elevated IOP, and (**B**) to five weeks of elevated IOP (n=40 in all experimental groups). Results are expressed as mean±SEM. Statistically significant differences were assessed according to Student’s *t*-test. The two asterisks denote the level of significance, p≤0.01. Abbreviations: intraocular pressure (IOP), protein kinase C-α (PKC-α).

The results show that after one week of elevated IOP, there was no significant decrease in the number of cells located in the innermost portion of the INL (Figure 6A). However, after five weeks of elevated IOP, there was a significant reduction in the number of cells located in the innermost portion of the INL (Figure 6B).

### Retinal ganglion cells

**Figure 7:**
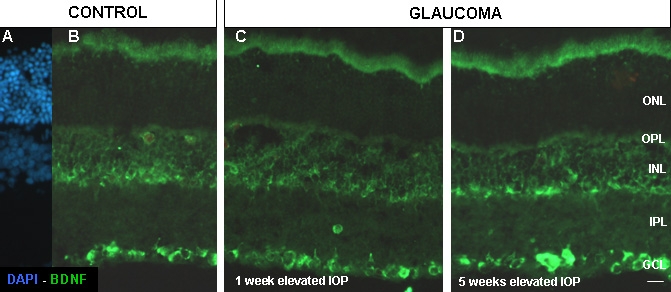
Fluorescent immunoreactivity of BDNF in control and glaucomatous retinas. Control retinas (**A** and **B**), and experimental glaucomatous retinas after one week (**C**) and five weeks (**D**) of elevated IOP. Abbreviations: brain derived neurotrophic factor (BDNF), ganglion cell layer (GCL), inner nuclear layer (INL), intraocular pressure (IOP), inner plexiform layer (IPL), outer nuclear layer (ONL), outer plexiform layer (OPL). Scale bar equals 20 μm.

Brain derived neurotrophic factor (BDNF) was expressed in amacrine and RGCs. There was no apparent difference in the labeling of BDNF between the control and glaucomatous retinas (Figure 7A-D).

### Glial cells

**Figure 8:**
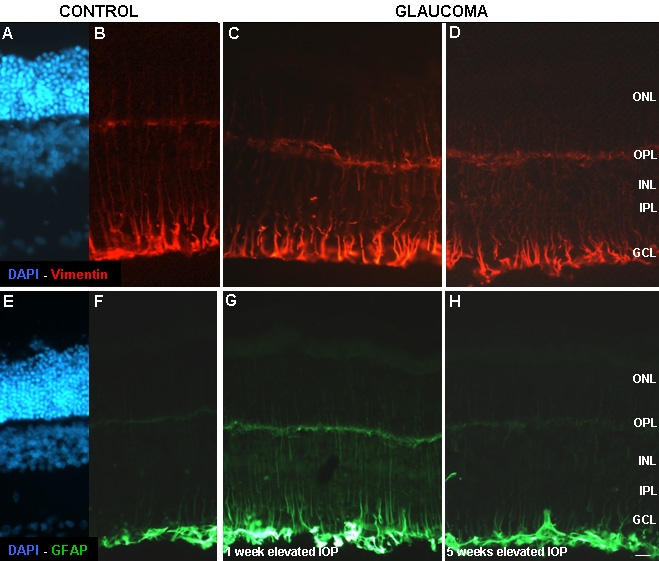
Fluorescent immunoreactivity of vimentin and GFAP in control and glaucomatous retinas. Panels **A-D** depict vimentin (red) antibody expression in control (**A-B**) and glaucomatous retinas after one week (**C**), and five weeks (**D**) of elevated IOP. Panels **E**-**H** indicate GFAP (green) antibody expression in astrocytes in control (**E**-**F**), one week (**G**), and five weeks of elevated IOP (**H**). Nuclei of the cells were marked with DAPI (blue). Abbreviations: ganglion cell layer (GCL), green fibrillary acidic protein (GFAP), inner nuclear layer (INL), intraocular pressure (IOP), inner plexiform layer (IPL), outer nuclear layer (ONL), outer plexiform layer (OPL). Scale bar represents 20 μm.

Figure 8 shows immunolabeling of the glial cells with vimentin and glial fibrillary acidic protein (GFAP) antibodies. Vimentin labels Müller cells processes, mainly in the inner limiting membrane of the retina (Figure 8B). The intensity of labeling of Müller cells and their end feet increased in group 1, compared to the controls (Figure 8C). However, the distribution and intensity of the vimentin positive Müller cells, in retinas after five weeks of elevated IOP, was similar to the control retinas (Figure 8D). Moreover, a disorganized pattern of Müller cells processes was visualized in the last group. 

GFAP labels astrocytes (Figure 8F). GFAP labeling in group 1 (Figure 8G) was more intense than the labeling in group 2 (Figure 8H).

## Discussion

Experimental rat glaucoma induced by episcleral vein cauterization produces a chronic ocular hypertension that leads to the progressive loss of RGCs [8]. However, early studies performed in human, monkey, or rodent spontaneous or experimental glaucoma, have not detected loss of any retinal cells other than RGCs. We report here variations in the expression of different molecular markers that identify subpopulations of retinal cells, in glaucomatous retinas. Our data reinforce the concept that although the critical pathology of glaucoma affects mostly RGCs, the disease has a profound impact on the whole retina.

In this study, we did not find any alterations in photoreceptor opsin expression when comparing the glaucomatous and control retinas. Similar results were found by other authors who observed no changes in rod and cones from patients suffering primary open angle glaucoma [28] or in animal models of experimental glaucoma [29,30]. A study in monkeys with glaucoma found a decrease in cone opsin mRNA levels [31]. This study had hypothesized that a reduced choroidal blood flow might result in a reduction of oxygenation of the photoreceptor cell layer in primates.

Horizontal cells were labeled with calbindin antibodies. No changes in the number of immunoreactive cells were found in glaucomatous retinas (Figure 3A-C). Similar results were reported in ischemia-reperfusion degenerative processes [32].

Calretinin labels AII amacrine cells in rabbits but PV antibodies labels AII amacrine cells in rat retina [33,34]. These markers also label other non-AII amacrine in monkey, rat, rabbit, and human retinas [35,36]. Five weeks after induced IOP elevation, we noted a decrease in the number and intensity of calretinin-positive amacrine cells in the INL. In ischemia-reperfusion experiments done in rat retinas, analogous results have been reported regarding calretinin immunostaining in amacrine cells [37].

PV labels AII amacrine cells, wide-field type amacrine cells, and displaced amacrine cells [34,38]. In the IPL, these interneurons are characterized by lobular appendages in sublamina a and narrow-field arboreal dendrites in sublamina b. The PV expression decreased in amacrine cells five weeks after elevation of IOP. These changes in the expression have not been reported before. PV-amacrine cells showed a reduction in immunoreactivity this could be due to the occurrence of a vigorous neurotransmitter release from their lobular appendages and to a passive opening of hemi-channels in arboreal dendrites of AII amacrine cells [39] or to a cell death.

ChAT antibody labeled those cholinergic amacrine cells that use acetylcholine as a neurotransmitter. Cholinergic amacrine cell bodies have been found in normal rat retinas. These cholinergic amacrine cells project their processes to strata 2 and 4 of the IPL [40]. In the present study, we have found fewer cholinergic amacrine cells after experimental induction of elevated IOP. The same observations in relation to the ChAT reduction have been described in glaucomatous rats after two to three weeks of elevated IOP [41], DBA/2J mouse retina [26,27] and in ischemic rat retina [22,23]. It is known that the function of RGCs is compromised when there is a loss of cholinergic signaling in the retina. In this sense, the loss of cholinergic amacrine neurons [42,43] or the loss of a neurotransmitter [28] has been demonstrated to cause changes in visual direction selectivity processing that could also be associated with glaucoma.

The alterations reported in the present study were mainly found in the inner retina. We observed immunohistochemical staining changes in cells labeled with PKC-α antibody. PKC-α is a molecular component of signal transduction pathways regulated by calcium. PKC-α-positive cells were localized in two rows of cells in the INL. The PKC-α-positive cells located in the innermost row disappeared in the glaucomatous retinas after five weeks of elevated IOP. We studied the possible colocalization of PKC-α with specific antibodies for amacrine cells in these cells. However, none of the markers studied colocalized with PKC-α in the PKC-α positive cells affected in glaucoma (data not shown). Therefore, we can not conclude that the cells were amacrine.

PKC-α antibody labels rod bipolar cells and dopaminergic amacrine cells in rat retinas [36,44]. However, we did not observe any decrease in the number of dopaminergic amacrine cells. Thus, we believe that some PKC-α-positive cells present in the control retinas did not express PKC-α or disappear in glaucomatous retinas.

To identify if the affected group of TH labeled cells that decreased in number in the glaucomatous retinas were amacrine cells, we did a double labeling with Goα. We found that the number of PKC-α-positive and Goα-negative cells decreased after five weeks of elevated IOP (Figure 6B), confirming that the type of affected cells could be either a subtype of amacrine positive for PKC-α but negative for calretinin, ChAT, or PV or a subtype of PKC-α bipolar cell.

AII amacrine cells represent the major output of rod bipolar cells. Signaling from rod and cone pathways converges in the inner retina, specifically in AII amacrine cells. In mammalian retinas, rod signal passes into the cone pathways by means of gap junctions established between AII amacrine cells and “on” cone bipolar cells [45].

We cannot confirm if the PKC-α rod bipolar cells that disappeared in glaucomatous retinas have died or are not expressing these molecular markers. Nonetheless, this alteration may be related to the changes noticed in ERG from glaucomatous rats [13]. It is assumed that OPs are generated by bipolar cells that make contact with amacrine cells and the dendrites of RGCs [46]. Our results suggest that glaucoma induces an extensive alteration in a subtype of amacrine cells and in rod and cone “on” bipolar cells that may become hypersensitive to the inputs from amacrine cells.

It is worth pointing out that not only were the amacrine cells affected by RGCs degeneration, but also the second order presynaptic bipolar neurons were affected. Interestingly, amacrine and bipolar cell damage has also been seen in animals with retinitis pigmentosa (P23H, RCS rats) [37,47] and Leber congenital amaurosis (RPE65 canine retina) [48]. However, in these diseases, cell damage initiates in the opposite part of the retina, starting with the photoreceptor cell layer and projecting toward the INL cells.

The distribution of BDNF-positive cells was not apparently affected. Although we did not count the number of cells in this study, we have previously demonstrated that after experimental glaucoma, RGCs die by apoptosis [5,8] and that all RGCs are positive to BDNF [49,50], thus we cannot disregard the observation that the elevation of IOP reduced the number of BDNF positive cells.

Glial cells have been shown to play an important role in the metabolism of neurotransmitters. In addition, they serve as modulators of synaptic transmission and help to maintain a homeostatic environment for neurons [51]. It is well documented that under normal conditions Müller cells do not express GFAP [52], being only present in astrocytes, while vimentin is the natural cytoskeleton component present in Müller cells. Different types of damage, including glaucoma, induce the expression of GFAP in Müller cells [41,53–55]. Our results confirm these observations. GFAP and vimentin immunoreactivity was markedly increased in Müller cells after one week of elevated IOP. Furthermore, after five weeks of elevated IOP, vimentin immunoreactivity showed a disorganized pattern of Müller cell extensions, similar to what we [50] and others [56] have found after acute ischemia.

Based on our results, we consider important to further investigate if a subpopulation of RGCs dies soon after IOP elevation. Also, the relationship of these RGCs with the circuitry of the rod and “on” cone pathways would be worth analyzing.

The changes observed in the distribution of the immunoreactivity in the hypertensive rat retina are more severe in the inner retina than in the outer retina, and especially affect the AII amacrine and bipolar cells. To our knowledge, we have described for the first time that the bipolar cells pathway is also damaged in the hypertensive eye, given the changes observed with the PKC-α and Goα antibodies. The alterations in bipolar cells are not surprising when one considers that RGCs lose their presynaptic connections. The changes noticed may represent a plasticity mechanism or a neuronal reprogramming in the circuitry [57]. The degeneration of the retina is a dynamic process where a loss of afferents induces changes in the connectivity and in the molecular expression of their cells.
